# Commentary: Do not forget to read history: You will understand and improve

**DOI:** 10.1016/j.xjtc.2021.09.007

**Published:** 2021-09-07

**Authors:** Carlos A. Mestres

**Affiliations:** a Department of Cardiac Surgery, University Hospital Zürich, Zürich, Switzerland; b Department of Cardiothoracic Surgery, The University of the Free State, Bloemfontein, South Africa

**Figure:**
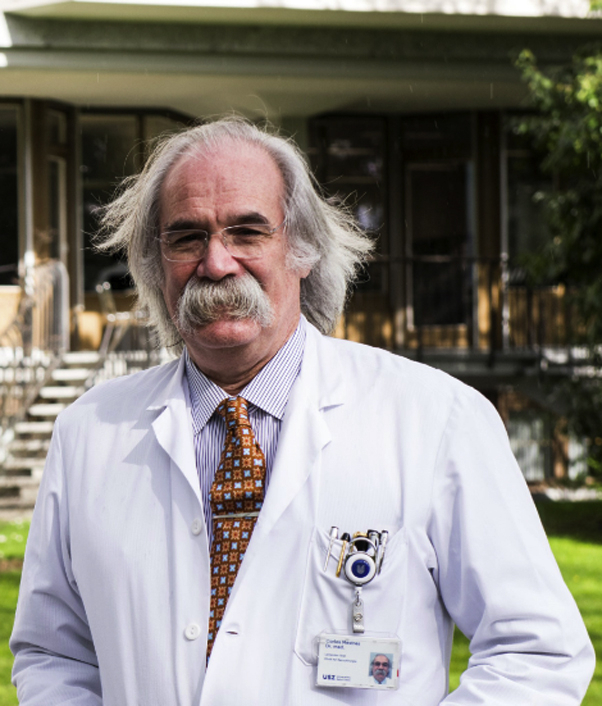
Carlos A. Mestres, MD

**Central Message**

This is an important historical vignette to understand the value of pioneering efforts in surgery and the impact of milestones on practice and outcomes. Operations in arrested hearts are currently routine worldwide.

“The important thing is to not stop questioning. Curiosity has its own reason for existing”

—Albert Einstein

See Article page 460.

The word *history* has several meanings. Sources define it as all the events that happened in the past, as past events connected with the development of a particular place or subject, as the study of past events, a written or spoken account of past events, or the set of facts that are known about someone's past life.^1^ This should give anyone a more or less broad perspective of what happened, for us to understand what is going on today, to learn from what was done aiming at avoiding possible mistakes, and to pave the way for our future, whichever it be.

The main question, then, is if we learned from the past, from history. There are many examples showing that we do not always learn the good things and that we often repeat mistakes. On the other side, reviewing history makes one understand that current policies, procedures, or acts are mostly based on what was done before, right or wrong. A problem is that many current graduates do not routinely read history, and many even question why the history of medicine is taught.^2^ Again, we will always learn and this is why we progressed from the past, when poor or no training, no scientific rigor, and nonexistent standards were the rule in medicine, to today's standards of evidence-based medicine and patient-centered care. By the way, remember that we owe the latter to William Osler.^3^

The brief historical vignette by our prestigious colleagues Drs Svensson and Mihaljevic^4^ published in this issue of the *Journal* on the first known stopped-heart operation is a good example of something done in the past having great impact on our current behavior and practice. Drs Effler and Groves at the Cleveland Clinic arrested the heart with potassium to conduct the intracardiac repair of a ventricular septal defect, something previously done by others without arrest, as referenced by the authors.^5^ Arresting the heart provided a still scenario and a bloodless field. The consequence is this approach is being used worldwide with success, resulting in hundreds of thousands of patients who have benefited from it for decades now. We need to understand how courageous all these pioneers were, with the heart stopped or not. We learned from them, we learned from history. Many of these patients survived for decades.^6^

We need to read history to know, understand, and improve. We need to read history, as its lessons are vitally relevant in science. Cross-circulation,^5^^,^^6^ the heart–lung machine,^7^ hypothermia,^8^ the arrested heart,^4^ blood transfusion,^9^ and many other aspects of our past and present practice belong to history. Reading and re-reading history is a must, even in times when recent research supports that some university students are not prepared to successfully deal with academic texts, which may limit academic training.^10^ Svensson and Mihaljevic have brought to us another elegant and invaluable vignette for surgeons and physicians to remember, understand and learn.^4^ Based on this, our future is bright.^11^, ^12^, ^13^
